# Co‐Encapsulation of Curcumin and Fucoxanthin With Multilayer Structural Nanoparticles: In Vitro Programmed Sequential Release Behavior and In Vivo Bioavailability Studies

**DOI:** 10.1002/fsn3.71923

**Published:** 2026-05-18

**Authors:** Luhui Wang, Mingqing Wang, Ling Lv, Changhu Xue

**Affiliations:** ^1^ Shandong Peanut Research Institute Qingdao China; ^2^ College of Food Science and Engineering Ocean University of China Qingdao China

**Keywords:** co‐delivery system, in vitro digestion model, multilayer structural nanoparticles, programmed sequential release, release behavior

## Abstract

Food‐grade co‐delivery systems with programmed sequential release properties allow for sequential delivery of two bioactives to different sites in the gastrointestinal tract (GIT), improving the bioavailability of the two bioactives in vivo, which has become an emerging field of research. In this paper, multilayer structural nanoparticles (MSNPs) possessing programmed sequential release properties were constructed on the basis of the structural design principle for the co‐encapsulation of curcumin (Cur) and fucoxanthin (FUC) with gliadin, carboxymethyl konjac glucomannan, and chitosan hydrochloride using the layer‐by‐layer self‐assembly technique. The programmed sequential release behavior was investigated by in vitro digestion models. It was demonstrated that Cur located in the outer layer of MSNPs relies on a concurrent release mechanism involving diffusion and erosion in simulated gastric fluid. The release of FUC located in the inner layer is primarily driven by diffusion. A large amount of Cur was released in simulated intestinal fluid and driven by the erosive mechanism. Further, FUC was released in large quantities, and its release was governed by erosion in simulated colonic fluid under the influence of β‐mannanase. This work reveals a programmed switch from diffusion to erosion as MSNPs transit through different GIT segments, which has rarely been addressed in previous co‐delivery systems. Notably, it was confirmed by in vivo animal models that MSNPs increased the bioavailability of the outer layer bioactives and reduced the biodistribution of the inner layer bioactives in the upper GIT. The in‐depth study of the programmed sequential release behavior is important for expanding the application of MSNPs in food‐grade co‐delivery systems.

## Graphical abstract

Programmed sequential release of MSNPs.

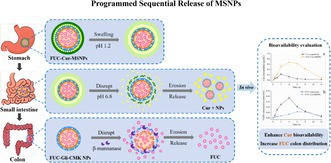

## 1 Introduction

The development of functional foods containing multiple bioactive components with synergistic health benefits has attracted increasing interest. It is well known that different bioactives often exert their physiological functions at distinct sites along the gastrointestinal tract (GIT). For example, curcumin (Cur) primarily acts in the small intestine (Rajasekaran 2011), where it exhibits anti‐inflammatory and antioxidant activities, whereas fucoxanthin (FUC) exerts its beneficial effects (e.g., anti‐cancer and anti‐obesity) mainly in the colon (Terasaki et al. 2019). Simply mixing two bioactives and delivering them together cannot achieve site‐specific release and optimal efficacy. Most currently investigated co‐delivery systems are single‐targeted for the GIT, with predominantly small intestine‐targeted or colon‐targeted delivery systems, which exert efficacy at a single site of the GIT. To address this challenge, co‐delivery systems that can sequentially release different bioactives at predetermined GIT sites are urgently needed. Nevertheless, most existing food‐grade co‐delivery systems are designed for single‐target release (either small intestine or colon), and only a few studies have attempted to construct programmed sequential release systems. Even in those reports, the release kinetics and underlying mechanisms remain poorly understood. In response to the current research status, we constructed multilayer structural nanoparticles (MSNPs) on the basis of the structural design principle, which were fabricated by layer‐by‐layer adsorption of carboxymethyl konjac glucomannan (CMK) with colon‐degradation performance (Wang et al. 2019, 2022) and chitosan hydrochloride (CHC) with excellent bioadhesive properties (Hua et al. 2023) on the surface of gliadin nanoparticles (Gli NPs). In our former study, MSNPs were prepared for co‐encapsulating Cur and FUC, and a series of experiments confirmed that MSNPs possessed programmed sequential release performances and were capable of sequentially delivering encapsulated bioactives to the small intestine and colon (Wang et al. 2023). Cur and FUC were selected as hydrophobic model bioactives targeting the small intestine and colon, respectively, and this multilayer structural design can be extended to other bioactive pairs with similar site‐specific requirements in the GIT. However, there was no in‐depth study on the changes of MSNPs during digestion, and the programmed sequential release behavior of the encapsulated bioactives, as well as their bioavailability in vivo, were not further investigated.

The release behaviors in the GIT of some novel complex food‐grade delivery systems constructed to date are not well defined and understood. The release of bioactives from delivery systems can involve multiple mechanisms, including diffusion, swelling, erosion, dissolution, permeation, and degradation (Rezaei et al. 2022). In‐depth study of the release behavior facilitates the design and construction of suitable delivery systems for encapsulating bioactives (Malekjani and Jafari 2022). The composition, structure, size, morphology, and swelling ability in gastrointestinal fluids of the delivery system, as well as the nature and concentration of the encapsulated bioactives, affect the release kinetics and release behavior (Teimouri et al. 2022). For food‐grade NPs, their composition exerts a dramatic influence on the release mechanism of the bioactives they encapsulate in the GIT. Depending on the unique properties of their carrier materials, NPs exhibit different release behaviors at different stages of gastrointestinal digestion (Zhou and McClements 2022). The dynamic processes of releasing bioactives from NPs‐based delivery systems are strongly linked to the swelling and disintegration of the materials used to construct the carriers in the oral, gastric, small intestinal, and colonic environments. In addition, the inherent properties of food‐grade NPs, including particle size, shape, and interfacial properties, also influence their release behavior in the GIT, which in turn alters the digestibility and bioavailability of the encapsulated bioactives (Pan and Zhong 2016; Moradi et al. 2022). Consequently, the use of in vitro digestion models to study the morphological changes, release, degradation or conversion into bioavailable forms of encapsulated bioactives throughout the GIT contributes to a better understanding of their digestion and release mechanisms. Furthermore, the use of in vivo animal models to assess the release and bioavailability of encapsulated bioactives is more conducive to developing and applying delivery systems (Chang et al. 2022).

To this end, MSNPs co‐encapsulating FUC and Cur (FUC‐Cur‐MSNPs) with ideal dispersibility and encapsulation properties were prepared based on our previous studies using Cur and FUC as model bioactives that exert beneficial effects on the small intestine (Rajasekaran 2011) and colon (Terasaki et al. 2019), respectively. Changes in particle properties and morphology of MSNPs were measured before and after digestion by simulated gastric fluid (SGF), simulated intestinal fluid (SIF), and simulated colonic fluid (SCF). The release behavior of MSNPs in different formulations of simulated gastrointestinal fluids was explored. In addition, the release kinetics and release mechanisms of MSNPs in simulated gastrointestinal fluids were evaluated by Zero‐order, First‐order, Higuchi, and Korsmeyer‐Peppas kinetic models. Lastly, the bioavailability of the encapsulated bioactives was evaluated by determining the pharmacokinetic parameters after gavage of MSNPs in mice, which served as the in vivo animal model. The present study systematically elucidates the programmed sequential release mechanisms of MSNPs and their in vivo bioavailability, thereby advancing the application of MSNPs in co‐delivering multiple bioactives with site‐specific requirements.

## 2 Materials and Methods

### 2.1 Materials

Fucoxanthin (FUC, purity ≥ 80%) was provided by Shandong Jiejing Group Co. Ltd. (Rizhao, China). Curcumin (Cur, purity ≥ 95%) was supplied by Shanghai Ryon Biological Technology Co. Ltd. (Shanghai, China). Gliadin (Gli, protein content 91.2%) and chitosan hydrochloride (CHC, DD 80%–90%, Mw 1.0 × 10^5^ Da) were provided by Shanghai Macklin Biochemical Co. Ltd. (Shanghai, China). Carboxymethyl konjac glucomannan (CMK, DS 0.55, Mw 4.8 × 10^5^ Da) was obtained as published previously (Wang et al. 2019).

### 2.2 Fabrication of FUC‐Cur‐MSNPs

**FIGURE 1:**
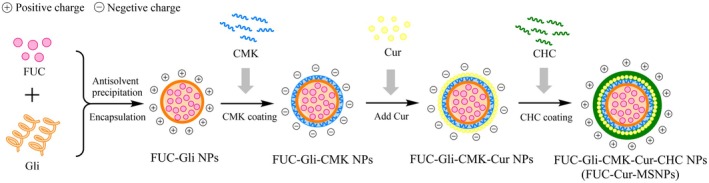
Preparation flow chart of FUC‐Cur‐MSNPs.

MSNPs co‐encapsulated FUC and Cur were prepared according to our previous study (Wang et al. 2023). At first, FUC‐Gli NPs were prepared by dispersing 20 mg/mL Gli and 2 mg/mL FUC in a 70% ethanol solution through the antisolvent precipitation method. The resulting mixture was added drop‐by‐drop at a volume ratio of 1:9 to pure water at pH 3.0 with continuous stirring. CMK solution at pH 3.0 was then added drop‐by‐drop in equal volume at a mass ratio of Gli/CMK of 1:1 to prepare FUC‐Gli‐CMK NPs. Following this, 1 mg/mL of Cur dissolved in 70% ethanol solution was added to FUC‐Gli‐CMK NPs at a volume ratio of 1:4, and stirred to fabricate FUC‐Gli‐CMK‐Cur NPs. Finally, an equal volume of 1 mg/mL CHC solution (pH 3.0) was added with continuous stirring to obtain FUC‐Gli‐CMK‐Cur‐CHC NPs (hereafter referred to as FUC‐Cur‐MSNPs). Figure 1 shows the preparation flow chart of FUC‐Cur‐MSNPs.

### 2.3 FUC‐Cur‐MSNPs Characterization

#### 2.3.1 Particle Size and ζ Potential

The particle size and ζ potential of FUC‐Cur‐MSNPs were detected using a dynamic light scattering instrument (Zetasizer Nano ZS 90, Malvern, UK) at 25°C. Before determination, FUC‐Cur‐MSNPs were diluted by water to avoid multiple scattering effects.

#### 2.3.2 Morphology

The morphology of FUC‐Cur‐MSNPs was observed by scanning electron microscopy (SEM). The freeze‐dried FUC‐Cur‐MSNPs were mounted on a sample holder and sprayed with gold. The SEM image was observed by a SEM (VEGA3, TESCAN Bruker, Germany) under the condition of an acceleration voltage of 20 kV.

#### 2.3.3 Bioactives Content Analysis

The content of bioactives (FUC and Cur) in FUC‐Cur‐MSNPs was measured via high‐performance liquid chromatography (HPLC). FUC and Cur were extracted by methanol after centrifugation at 4000 *g* for 20 min, and the methanol extracts were filtered through 0.22 μm filters. The following chromatographic conditions were used. For FUC, the column was equilibrated and isocratically eluted with a mixture of 10% water and 90% methanol at a flow rate of 1.0 mL/min and 30°C (Hashimoto et al. 2009), and eluents were assessed at a wavelength of 450 nm. For Cur, the gradient elution was carried out with the mobile phase consisting of 4% (v/v) acetic acid (A) and acetonitrile (B) (0.01–10 min, 30%–100% (B), 10.01–15 min, 100%–30% (B)) (Wichitnithad et al. 2009). The column temperature was set at 25°C, the wavelength was 425 nm, and the flow rate was 1.0 mL/min. All chromatographic separations were achieved using a C18 analytical column (150 × 4.6 mm) with 5 μm particle size.

### 2.4 Release Behavior of MSNPs at Simulated Gastrointestinal Conditions

In vitro simulated digestions of FUC‐Cur‐MSNPs in SGF, SIF, and SCF were conducted according to previous methods (Wang et al. 2023) with some modifications. SGF (pH 1.2) was prepared by dissolving 9600 U/L pepsin in hydrochloric acid solution. SIF (pH 6.8) was prepared by dissolving enzymes (25,000 U/L trypsin, 1.6 mg/mL lipase, 10 U/mL α‐amylase) and 5 mg/mL bile salts in phosphate buffer solution (PBS). SCF (pH 7.4) was prepared by dissolving 6000 U/L β‐mannanase in PBS.

A quantity of freeze‐dried FUC‐Cur‐MSNPs (10 mg) was dispersed in 5 mL SGF to simulate the gastric digestion process at 37°C with shaking. Samples were taken at intervals of 0.5 h and centrifuged at 4000 *g* for 20 min, and the collected supernatants were assayed for released bioactives according to method 2.3.3. Following each centrifugation, FUC‐Cur‐MSNPs were redispersed in fresh simulated gastrointestinal fluid for digestion until the follow‐up sampling. After digestion in SGF for 2 h, FUC‐Cur‐MSNPs were dispersed in 5 mL of SIF to simulate the intestinal digestion process, and sampling was performed. After digestion in SIF for 3 h, FUC‐Cur‐MSNPs were redispersed in 5 mL of SCF to simulate the colon digestion process, and the above sampling operation was repeated. The amount of bioactives released during the simulated digestion was calculated, and the cumulative release was quantified based on the total bioactives initially added to the FUC‐Cur‐MSNPs. Additionally, the particle size, size distribution and ζ potential of FUC‐Cur‐MSNPs after digestion in SGF, SIF and SCF were determined according to method 2.3.1. The morphology changes of FUC‐Cur‐MSNPs after digestion with simulated gastrointestinal fluids were observed following freeze‐drying, in accordance with the procedure in Section 2.3.2.

### 2.5 In Vitro Release Kinetics

Release kinetics of MSNPs were investigated by fitting the release data of Cur and FUC in simulated gastrointestinal fluids with Zero‐order, First‐order, Higuchi, and Korsmeyer‐Peppas release kinetic models (Xiao et al. 2021). The model with the highest correlation coefficient (*R* ^2^) was judged to be the optimal kinetic model for Cur and FUC release in MSNPs. The formulae are described below.

(1) Zero‐order model:Mt/M∞=kt

(2) $$\text{First}‐\text{order model}:\ln\left(1-\mathrm{Mt}/M\infty \right)=-\mathrm{kt}$$

(3) $$\text{Higuchi model}:\mathrm{Mt}/M\infty ={\mathrm{kt}}^{1/2}$$

(4) $$\text{Korsmeyer}‐\text{Peppas model}:\mathrm{Mt}/M\infty ={\mathrm{kt}}^{n}$$

in which *Mt*/*M∞* is the cumulative release amount of bioactives at *t*, *k* is the rate constant, and *n* is the diffusional exponent.

### 2.6 In Vivo Bioavailability Evaluation

After 1 week of acclimatization, 4‐week‐old male ICR mice were divided into three groups and gavaged with FUC suspension, Cur suspension, and FUC‐Cur‐MSNPs (10 mg/kg FUC body weight, 25 mg/kg Cur body weight), respectively. Subsequently, all the mice were allowed food and water ad libitum. Blood samples were obtained from the eyeballs of mice (six mice per group) into EDTA‐containing microtubes at 0.5, 1, 2, 4, 6, 8, and 12 h after oral gavage, and centrifuged (4°C, 5000 rpm, 10 min) to collect plasma (Wang et al. 2021). Ethical approval was obtained by the Animal Ethics Committee of Ocean University of China.

To extract Cur, 100 μL of plasma was added to 250 μL ethyl acetate, vortexed for 60 s, and the supernatant was collected after centrifuging (4°C, 10,000 rpm, 5 min). For the extraction of FUC and its metabolites, 400 μL of dichloromethane/methanol (1:2, v/v) was mixed with 100 μL of plasma and vortexed for 60 s. Next, 200 μL of n‐hexane was added to the mixture and vortexed for 30 s. The mixture was then centrifuged (4°C, 1000 *g*, 5 min), and the resulting upper phase was collected (Li et al. 2021). All the above extraction processes were repeated twice. The combined extracts were blow‐dried with nitrogen, redissolved in 100 μL of methanol, and detected after filtration. The plasma concentration of Cur or FUC was determined using an HPLC system according to method 2.3.3.

### 2.7 Statistical Analysis

Data were expressed as the mean value ± standard deviation. Statistical analysis was performed using one‐way analysis of variance (one‐way ANOVA), and *p* < 0.05 was considered statistically significant.

## 3 Results and Discussion

### 3.1 Characterization of FUC‐Cur‐MSNPs in Simulated Gastrointestinal Conditions

**FIGURE 2:**
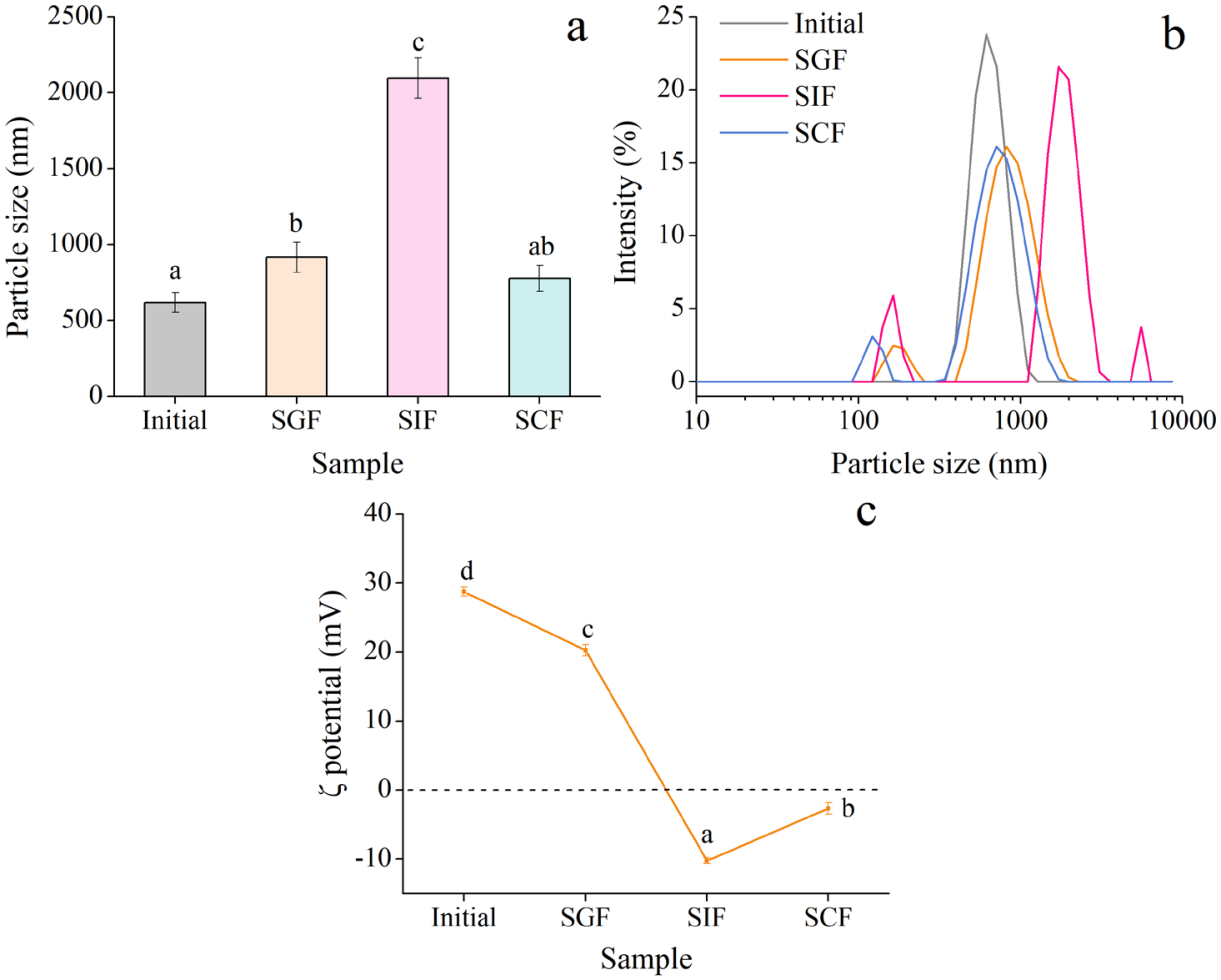
Particle size (a), size distribution (b), and ζ potential (c) of FUC‐Cur‐MSNPs in simulated gastrointestinal fluids.

The change in particle properties of NPs‐based delivery systems in simulated gastrointestinal fluids is an important indicator for evaluating their stability in the GIT (Hörter and Dressman 2001). Variations in particle size, size distribution, and ζ potential of FUC‐Cur‐MSNPs in simulated gastrointestinal conditions are presented in Figure 2. The initial undigested MSNPs displayed a small particle size of about 618.2 nm with a single size distribution, and the initial FUC‐Cur‐MSNPs are positively charged due to the CHC coating. The particle size of FUC‐Cur‐MSNPs tended to increase to 916.5 nm after 2 h of digestion in SGF, which could be attributed to the swelling of their outermost carrier material, CHC, and the disruption of the structure of a small amount of FUC‐Cur‐MSNPs led to the aggregation of exposed FUC‐Gli‐CMK‐Cur NPs and free Cur. This swelling behavior under acidic conditions is consistent with the structural alterations of polysaccharide‐coated protein NPs reported by Chang et al. (2017), who observed that pectin‐coated caseinate/zein NPs also exhibited significant size expansion in SGF due to hydration and electrostatic repulsion attenuation. The positive charge of MSNPs decreased due to electrostatic shielding between the particles caused by the high ionic strength under the acidic pH conditions of SGF. In addition, the decrease in the positive charge of FUC‐Cur‐MSNPs was also associated with a small amount of desorption of their outermost layer of CHC in SGF. Notably, FUC‐Cur‐MSNPs exhibited resistance to pepsin digestion owing to the protective effect of the outermost layer of polysaccharides, and MSNPs were able to maintain their relative integrity in SGF compared to protein NPs (Li et al. 2023). The particle size of FUC‐Cur‐MSNPs increased significantly, and the size distribution became uneven after 3 h of digestion in SIF, showing a multi‐peak particle size distribution as well as several small particles, whose charges changed from positive to negative. On the one hand, the environment of SIF led to further swelling of MSNPs and partial desorption of CHC from the surface of MSNPs, exposing a large number of negatively charged NPs. The complex system contained free Cur, FUC‐Gli‐CMK NPs, FUC‐Gli‐CMK‐Cur NPs, and other unstable polyelectrolytes, which aggregated, resulting in an increased particle size. On the other hand, it has been shown that lipid‐soluble bioactives (e.g., Cur) released from delivery systems interact with bile salts and other components present in the small intestine, causing their recrystallization and adsorption onto the surface of the hydrophobic core to form smaller‐sized particles (Zou et al. 2016). Zou et al. (2016) demonstrated that Cur released from lipid‐based emulsions could form crystalline complexes with bile salts, yielding particle aggregates below 200 nm. Consistently, the particle size distribution of FUC‐Cur‐MSNPs after SIF digestion in this study revealed several small particles, suggesting analogous recrystallization events occurring at the hydrophobic core interface. Further, the size distribution profile of FUC‐Cur‐MSNPs changed after digestion in SCF with a decrease in particle size and negative charge carried, which can be explained by the gradual degradation of exposed FUC‐Gli‐CMK NPs in the colonic environment.

**FIGURE 3:**
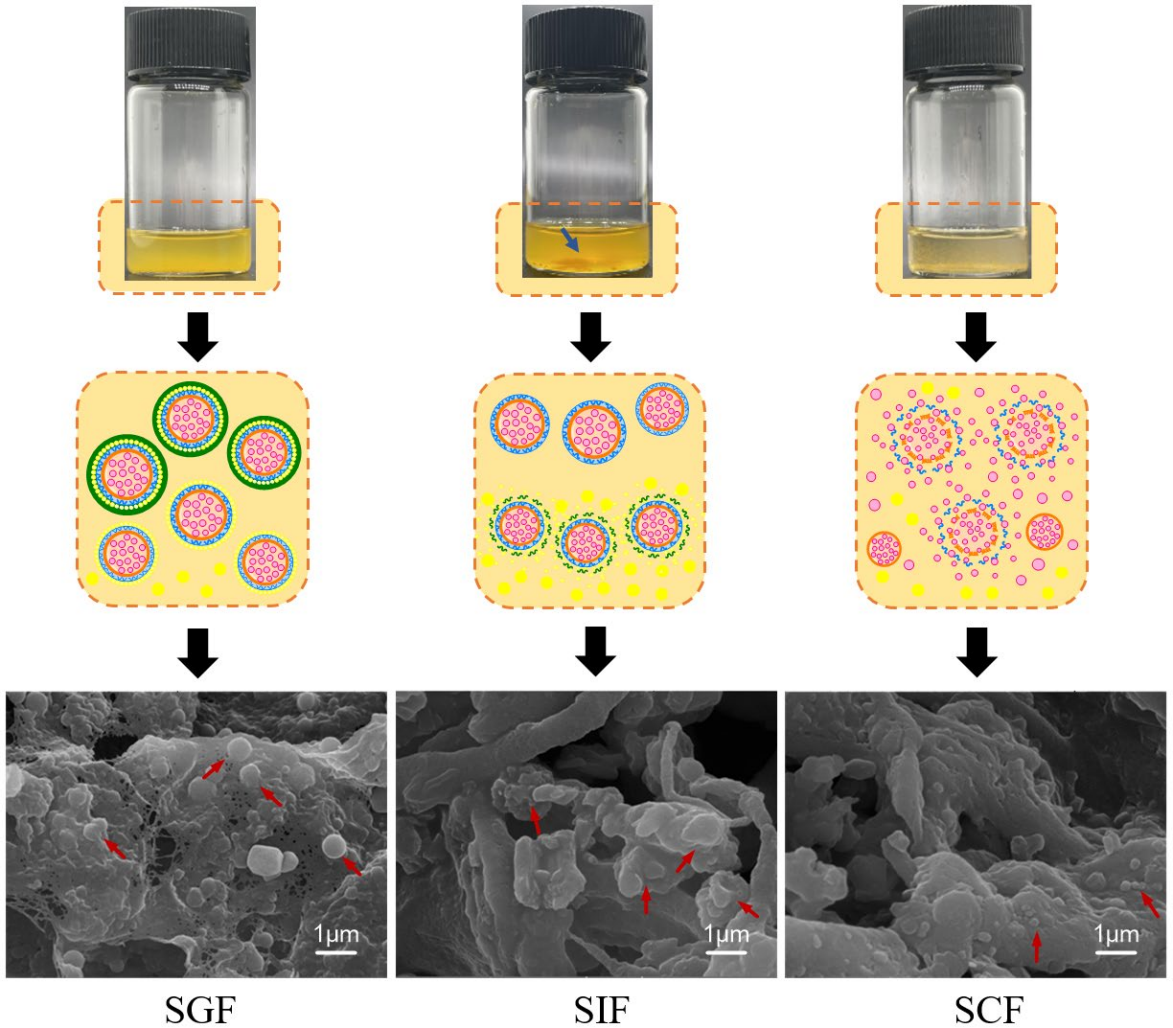
Morphology of FUC‐Cur‐MSNPs in simulated gastrointestinal fluids.

The morphology of FUC‐Cur‐MSNPs in simulated gastrointestinal fluid is displayed in Figure 3. FUC‐Cur‐MSNPs digested by SGF were more uniform, and no obvious precipitation appeared at the bottom, indicating that most of FUC‐Cur‐MSNPs were more stable in SGF and still maintained their initial state. SEM images could also corroborate that the vast majority of FUC‐Cur‐MSNPs remained intact, with only a minor number swelling or rupturing. The color of FUC‐Cur‐MSNPs digested by SIF was deepened, and the yellow precipitate was observed at the bottom. Mutual aggregation among multiple spherical particles could also be observed from the SEM image, which was consistent with the results of the particle sizes shown in Figure 2a. It was hypothesized that the outermost CHC layer of MSNPs in the SIF environment was swollen, and the released Cur, FUC‐Gli‐CMK NPs, and FUC‐Gli‐CMK‐Cur NPs present in the system might aggregate. Compared with digestion in SIF, the precipitation in the MSNPs system after SCF digestion was reduced, with fine suspended particles, and no obvious large spherical particles were found in the SEM images, indicating that the multilayers of MSNPs were gradually decomposed, and the exposed inner NPs were degraded. Presumably, the system primarily consisted of decomposed carrier materials, a small amount of undegraded particles, and released bioactives at this time, which weakened the aggregation between particles. This phenomenon is similar to the findings of pH‐responsive NPs prepared by Liang et al. (2022) using microfluidic technology for encapsulation and colon‐targeted release of FUC. In their study, aggregation and settlement of NPs at the bottom were observed after digestion in SGF and SIF. In contrast, no aggregation state of the particles was observed in SCF, and the system color changed significantly, suggesting that the prepared pH‐responsive NPs aggregated in SGF, dissociated in SIF, and completely degraded in SCF for targeted delivery of FUC.

### 3.2 Release Profiles of FUC‐Cur‐MSNPs in Simulated Gastrointestinal Conditions

**FIGURE 4:**
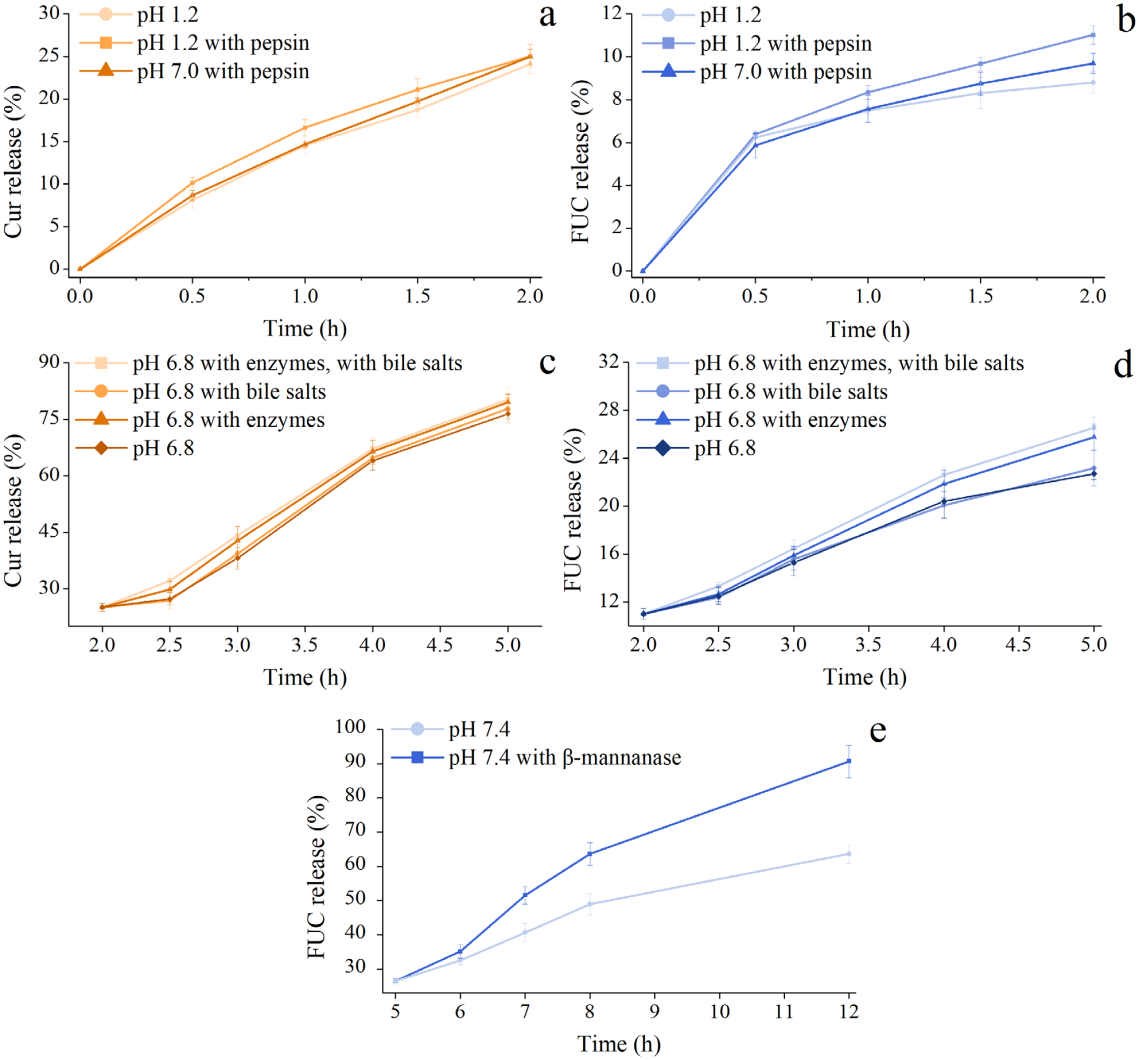
Effects of SGF (a, Cur; b, FUC), SIF (c, Cur; d, FUC), and SCF (e, FUC) on the release behavior of Cur and FUC in FUC‐Cur‐MSNPs.

The in vitro digestion model with different formulations of simulated gastrointestinal fluids was utilized to further explore the GIT factors affecting the programmed sequential release behavior of MSNPs. The effects of SGF on the release behavior of Cur and FUC in FUC‐Cur‐MSNPs are depicted in Figure 4a,b. After 2 h of digestion in different formulations of SGF, there was no significant difference in the rate of Cur release from MSNPs (Figure 4a), which was around 25%, suggesting that the acidic environment and pepsin of SGF did not affect the release behavior of Cur. CHC, as the outermost carrier material of MSNPs, was able to resist low pH and pepsin. It is therefore tempting to speculate that the structural changes of MSNPs in SGF might be due to the swelling of CHC rather than the pH environment and pepsin erosion (Tahir et al. 2025). This acid‐resistant property of chitosan‐based coatings is consistent with the findings of Yang et al. (2022), who demonstrated that hydroxypropyl trimethyl ammonium chloride chitosan‐modified solid lipid NPs maintained their structural integrity and exhibited prolonged release in SGF. However, unlike their system, which relied solely on chitosan derivatives for gastric protection, FUC‐Cur‐MSNPs constructed in this study employed a multilayer structure where CHC not only resisted acidic erosion but also served as a sacrificial swelling layer that preserved the integrity of inner NPs. Apparently, FUC released from MSNPs in SGF was low, only up to 11%, and the release behavior of FUC located in the innermost layer of MSNPs did not show remarkable differences with the composition of SGF (Figure 4b). It is hypothesized that the small amount of FUC released in SGF is attributed to the diffusion of FUC attached to the FUC‐Cur‐MSNPs surface as well as the cleavage of incompletely coated FUC‐Gli NPs. This surface‐associated release phenomenon has been systematically characterized by Sorasitthiyanukarn et al. (2024) in FUC‐loaded alginate/chitosan NPs, where approximately 15% of encapsulated FUC was released in SGF due to desorption of superficially adsorbed bioactive molecules. Their work corroborates our hypothesis that the initial FUC release originates from surface‐bound fractions rather than structural failure of the multilayer shell.

Influences of SIF on the release behavior of Cur and FUC in FUC‐Cur‐MSNPs are shown in Figure 4c,d. Similar to SGF, different formulations of SIF had no significant effect on the release behavior of Cur (Figure 4c). The Cur release reached approximately 78% after 3 h of digestion in different formulations of SIF, indicating substantial Cur release in the small intestinal environment. Furthermore, enzymes (including trypsin, lipase and amylase) and bile salts in SIF exerted minimal influence on the structure of MSNPs, which is consistent with the findings of Chen et al. (2020). Compared with the interaction of lipase and bile salts in SIF with lipids in the emulsion, the composition of SIF showed less impact on the release behavior of the encapsulated bioactives in NPs because of the absence of lipid components in NPs, speculating that the extensive release of Cur encapsulated in MSNPs in SIF may mainly rely on the swelling and diffusion of the outermost CHC layer. This enzyme‐independent release mechanism stands in marked contrast to the behavior of starch‐based NPs reported by Zhang et al. (2025), where digestive enzyme coronas (trypsin and lipase) dramatically modulated quercetin release kinetics through bile salt‐mediated corona formation. It further highlights the unique release mechanism of polysaccharide‐coated MSNPs compared to enzyme‐sensitive biopolymer carriers. Based on the above results, it was evident that enzymes in SIF had no meaningful effect on the Cur release from MSNPs. Therefore, the influence of a single factor, such as trypsin, lipase, and amylase, was not further explored in depth. As can be seen from Figure 4d, compared to the enzyme‐ and bile salts‐added SIFs, the release amount of FUC from FUC‐Cur‐MSNPs was reduced after digestion in the enzyme‐unadded SIF (pH 6.8 with bile salts), while the absence of bile salts (pH 6.8 with enzymes) did not alter FUC release in SIF. The results indicated that bile salts did not affect the release behavior of FUC encapsulated by MSNPs, whereas the influence of enzymes in SIF on FUC release was related to their degradation of the small amount of incompletely coated Gli NPs in the system. The negligible role of bile salts in MSNPs is noteworthy when contrasted with classical lipid‐based formulations. Early studies by Lairon et al. (1980) established that bile salts were indispensable for pancreatic lipase hydrolysis of monomolecular lipid films through colipase‐mediated binding. The insensitivity of the MSNPs constructed in this study to bile salts, therefore, reflects the non‐lipidic nature of the carrier matrix.

Figure 4e illustrates the effect of β‐mannanase in SCF on the FUC release from FUC‐Cur‐MSNPs. FUC was massively released in β‐mannanase‐added SCF. However, compared with the β‐mannanase‐added SCF, digestion in the β‐mannanase‐free SCF resulted in a dramatic reduction in the release amount and a marked slowdown in the release rate of FUC from FUC‐Cur‐MSNPs, confirming that β‐mannanase exerts an essential role in the FUC release of MSNPs. The multilayer structure of MSNPs was gradually destroyed after SGF and SIF digestion, and the β‐mannanase in SCF degraded the exposed carrier material, CMK, releasing the encapsulated bioactives (Zhang et al. 2014). It is inferred that the FUC located in the inner layer of MSNPs primarily relies on the erosive release of β‐mannanase. The β‐mannanase‐triggered colonic release mechanism aligns with the foundational work of Gliko‐Kabir et al. (2000), who demonstrated that crosslinked guar gum hydrogels retained their enzymatic degradability in the rat cecum, with β‐mannanase and α‐galactosidase activity mediating hydrocortisone release. FUC‐Cur‐MSNPs in this study exhibited an analogous enzyme‐responsive profile. However, the MSNPs achieved rapid FUC release within a relatively shorter timeframe in the presence of β‐mannanase. This accelerated release kinetics may stem from nanoscale structures and the partial pre‐degradation of the outer layer during gastrointestinal transit, rendering the CMK core more susceptible to enzymatic attack, which is a structural advantage absent in the hydrogel matrix.

### 3.3 Release Kinetics and Mechanism of FUC‐Cur‐MSNPs

**FIGURE 5:**
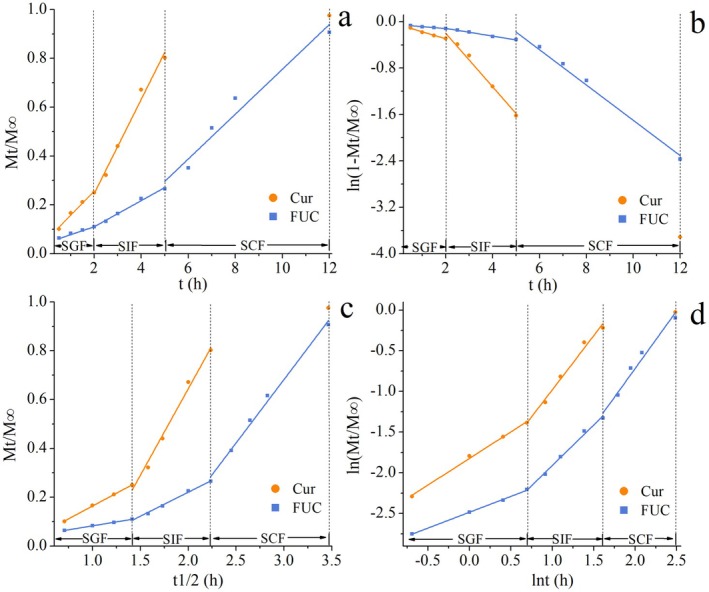
Kinetics fitting curves of Cur and FUC releases from FUC‐Cur‐MSNPs in simulated gastrointestinal fluids (a, Zero‐order model; b, First‐order model; c, Higuchi model; d, Korsmeyer‐Peppas model).

In order to clarify the programmed sequential release mechanism of MSNPs for the encapsulated two bioactives, Zero‐order, First‐order, Higuchi, and Korsmeyer‐Peppas kinetic models were employed to fit the release data of Cur and FUC in SGF, SIF, and SCF. The release mechanisms were obtained by characterizing the kinetic parameters of the models. The kinetics fitting curves and kinetic parameters of Cur and FUC released from FUC‐Cur‐MSNPs in simulated gastrointestinal fluids are presented in Figure 5 and Table 1, respectively.

**TABLE 1:** Kinetic parameters of Cur and FUC releases from FUC‐Cur‐MSNPs in simulated gastrointestinal fluids.

	Model		SGF	SIF	SCF
Cur	Zero‐order model	*k*	0.09831	0.19293	—
		*R* ^2^	0.97893	0.98429	—
	First‐order model	*R* ^2^	0.98739	0.98154	—
	Higuchi model	*R* ^2^	0.99920	0.98689	—
	Korsmeyer‐Peppas model	*n*	0.65271	1.32993	—
		*R* ^2^	0.99613	0.98594	—
FUC	Zero‐order model	*k*	0.03243	0.05351	0.09183
		*R* ^2^	0.96471	0.99026	0.95404
	First‐order model	*R* ^2^	0.98767	0.99282	0.98381
	Higuchi model	*R* ^2^	0.99867	0.99264	0.99136
	Korsmeyer‐Peppas model	*n*	0.38885	0.99383	1.41261
		*R* ^2^	0.99834	0.99317	0.96692

The release rate of MSNPs is dependent on the Zero‐order model, with a high *R* ^2^ value above 0.95. The *k* value is defined as an indicator of the release rate of the encapsulated bioactives, with a higher *k* value indicating a faster release of the bioactives from the delivery system (Fan et al. 2020). For the release of Cur from MSNPs, the *k* value of Cur in SIF was higher than that of SGF, and the faster release of Cur in SIF was associated with the complete swelling of the outermost CHC layer of MSNPs in SIF. Moreover, the *k* value of FUC in SCF was significantly larger compared with those of SGF and SIF, demonstrating the rapid release of FUC encapsulated by MSNPs in SCF, which is attributable to the degradation of exposed FUC‐Gli‐CMK NPs by β‐mannanase.

To deeply explore the release mechanism of MSNPs, the release data of co‐encapsulated Cur and FUC in simulated gastrointestinal fluids were fitted using the Korsmeyer‐Peppas model. The Korsmeyer‐Peppas model describes the release mechanism with more than one type of release phenomenon, such as diffusion, erosion, or both, by evaluating the *n* value. For spherical particles, *n* < 0.43 reflects a Fickian release case I transport, showing a diffusion release mechanism. Meanwhile, 0.43 < *n* < 0.85 reflects a non‐Fickian release, describing the concurrent release mechanism of diffusion and erosion. Additionally, *n* > 0.85 reflects a Fickian release case II transport, showing an erosion release mechanism (Ma et al. 2021). The release data of both Cur and FUC in simulated gastrointestinal fluids fitted well with the Korsmeyer‐Peppas model, and the *R* ^2^ values were both greater than 0.96. This goodness‐of‐fit (*R* ^2^ > 0.96) is comparable to the Korsmeyer‐Peppas model fitting results reported by Hussain et al. (2024) for chitosan‐derived carbon dots, where the *R* ^2^ value exceeded 95% and the release mechanism was confirmed as anomalous (non‐Fickian) transport. However, unlike their system which exhibited uniform anomalous transport across pH conditions, MSNPs displayed programmable shift in release mechanism, demonstrating the superior programmed sequential release afforded by multilayer structural design. The *n* value of Cur in SGF was in the range of 0.43–0.85, indicating that Cur encapsulated in MSNPs was attributed to the concurrent release mechanism of diffusion and erosion in SGF, which followed non‐Fickian diffusion. The *n* value of Cur in SIF was greater than 0.85, reflecting the case II transport mechanism, suggesting that the release of Cur in SIF was mainly based on erosion. In the case of FUC, its *n* value in SGF was less than 0.43, implying a Fickian release case I transport, when the release mechanism of FUC is mainly ascribed to diffusion. It is clear that the *n* values of FUC in SIF and SCF were higher than 0.85, showing that the innermost layer of FUC released from MSNPs during simulated small intestinal and simulated colonic digestion was governed by erosion.

Bioactives encapsulated in the delivery system are released by different mechanisms depending on the simulated gastrointestinal conditions. In general, the bioactives attached to the surface of the delivery system firstly diffuse into the simulated digestive fluids, leading to their initial phase of sudden release. Afterwards, the digestive fluids penetrate the swelling matrix, where the encapsulated bioactives undergo dissolution and diffusion. Ultimately, the swelling matrix is erosive, accompanied by the release of the remaining bioactives (Xiao et al. 2021). In this study, MSNPs were used as delivery carriers for bioactives, and the carrier materials, CHC and CMK, could be swollen and eroded in simulated gastrointestinal fluids. Simulated gastrointestinal fluids penetrated the outer layer and entered the MSNPs to induce their swelling, at which time the molecular chains became intertwined with each other. Cur and FUC attached to surfaces or encapsulated in MSNPs are susceptible to diffusion and release through macromolecular chains. Due to the protection of the carrier material, the encapsulated Cur and FUC diffused outward and released slowly in SGF. With the gradual dissolution of the carrier material in SIF, the MSNPs transformed into a relaxed state, and Cur located in the outer layer diffused through the swelling layer and was released by erosion. This swelling‐diffusion‐erosion transition mechanism is consistent with the in vitro digestion behavior observed by Wong et al. (2021) in alginate‐chitosan hydrogel beads encapsulating thymoquinone‐loaded Pickering emulsions. Their system exhibited low water uptake (19%) in SGF, followed by high swelling degree (85%) and superior water uptake (593%) in SIF, leading to erosion‐dominated release of thymoquinone (up to 83%) via bead swelling and diffusion processes. In SCF, the erosion of exposed Gli‐CMK NPs led to substantial release of FUC located in the inner layer.

Before further discussing the release mechanism, it is worth explaining why separate nanoparticles (Cur‐CHC NPs, FUC‐Gli‐CMK NPs) or their simple mixture were not used as controls. In our preliminary experiments, Cur‐CHC NPs alone exhibited a relatively low Cur encapsulation efficiency (approximately 52%) compared with that achieved in MSNPs (approximately 73%), and the physical mixture of positively charged Cur‐CHC NPs and negatively charged FUC‐Gli‐CMK NPs immediately aggregated due to electrostatic attraction, making the mixture unsuitable for reliable comparison. Moreover, our previous study (Wang et al. 2023) already demonstrated that MSNPs outperform FUC‐Gli‐CMK NPs alone in encapsulation efficiency, stability, and colon‐targeted delivery of FUC. Therefore, the physical mixture does not represent a meaningful control, and the present study focuses on elucidating the programmed sequential release mechanism of the MSNPs.

**FIGURE 6:**
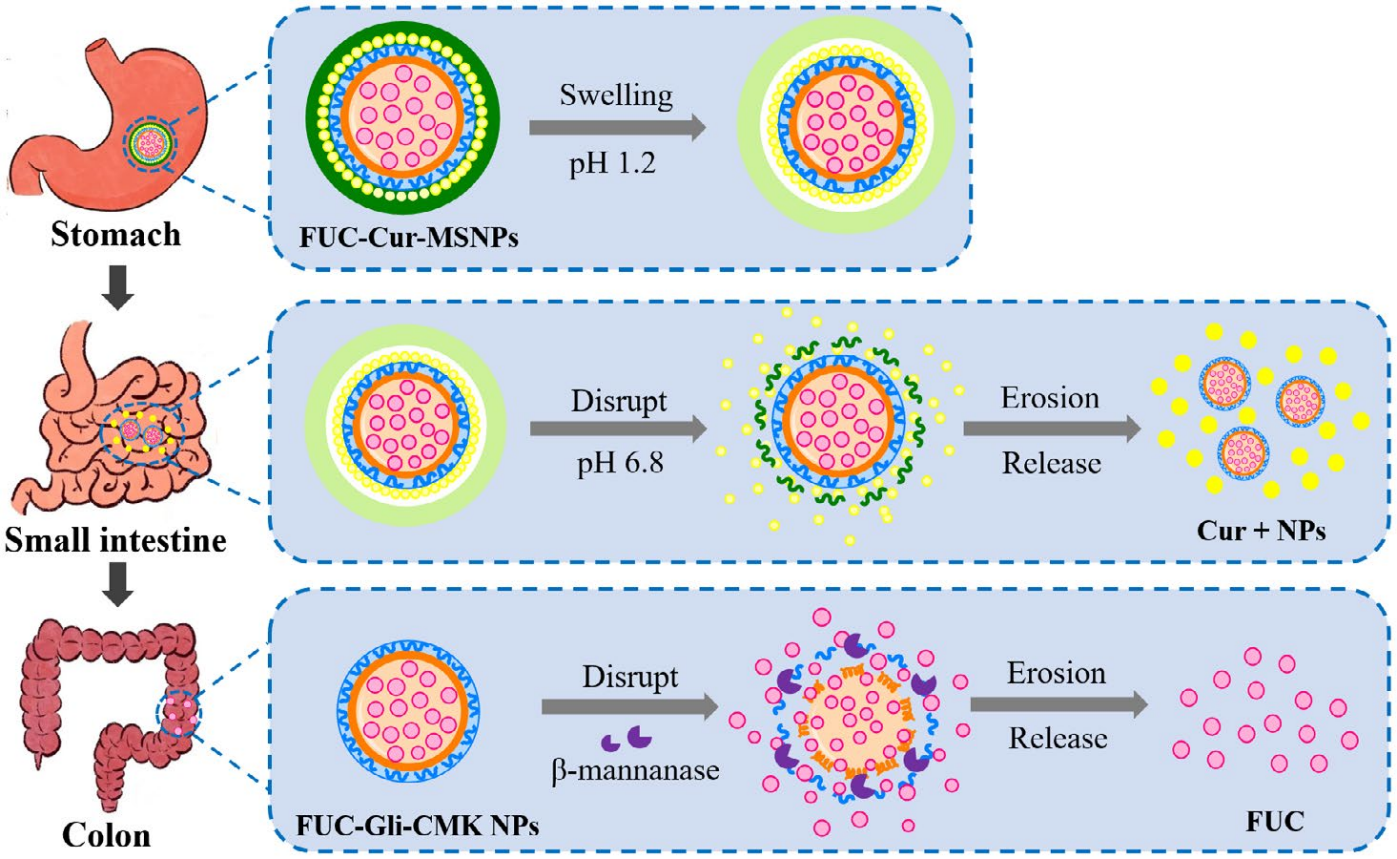
Schematic representation of the release mechanism of Cur and FUC in FUC‐Cur‐MSNPs.

Combined with all findings, the schematic illustration of the programmed sequential release mechanism of the two bioactives co‐encapsulated in MSNPs is assumed as shown in Figure 6. MSNPs can remain relatively stable in the gastric environment with less release of their co‐encapsulated Cur and FUC, where Cur located in the outer layer of MSNPs relies on the concurrent release mechanism of diffusion and erosion. In contrast, the release of FUC located in the inner layer is dominated by the diffusion mechanism. Acidic conditions of the stomach and the presence of pepsin exhibited no significant effect on the release of the co‐encapsulated bioactives. Upon transit to the small intestine, the outer CHC layer is swollen, and the structure of MSNPs is progressively disrupted. A large amount of Cur encapsulated in MSNPs is released by the erosive mechanism, which is insignificantly affected by the presence of enzymes and bile salts in the small intestine. In contrast, FUC is released relatively minimally in this environment. Due to the action of β‐mannanase in the colonic environment, FUC located in the innermost layers of MSNPs is massively released upon arrival in the colon, predominantly through erosion.

### 3.4 Pharmacokinetics Analysis of FUC‐Cur‐MSNPs

**FIGURE 7:**
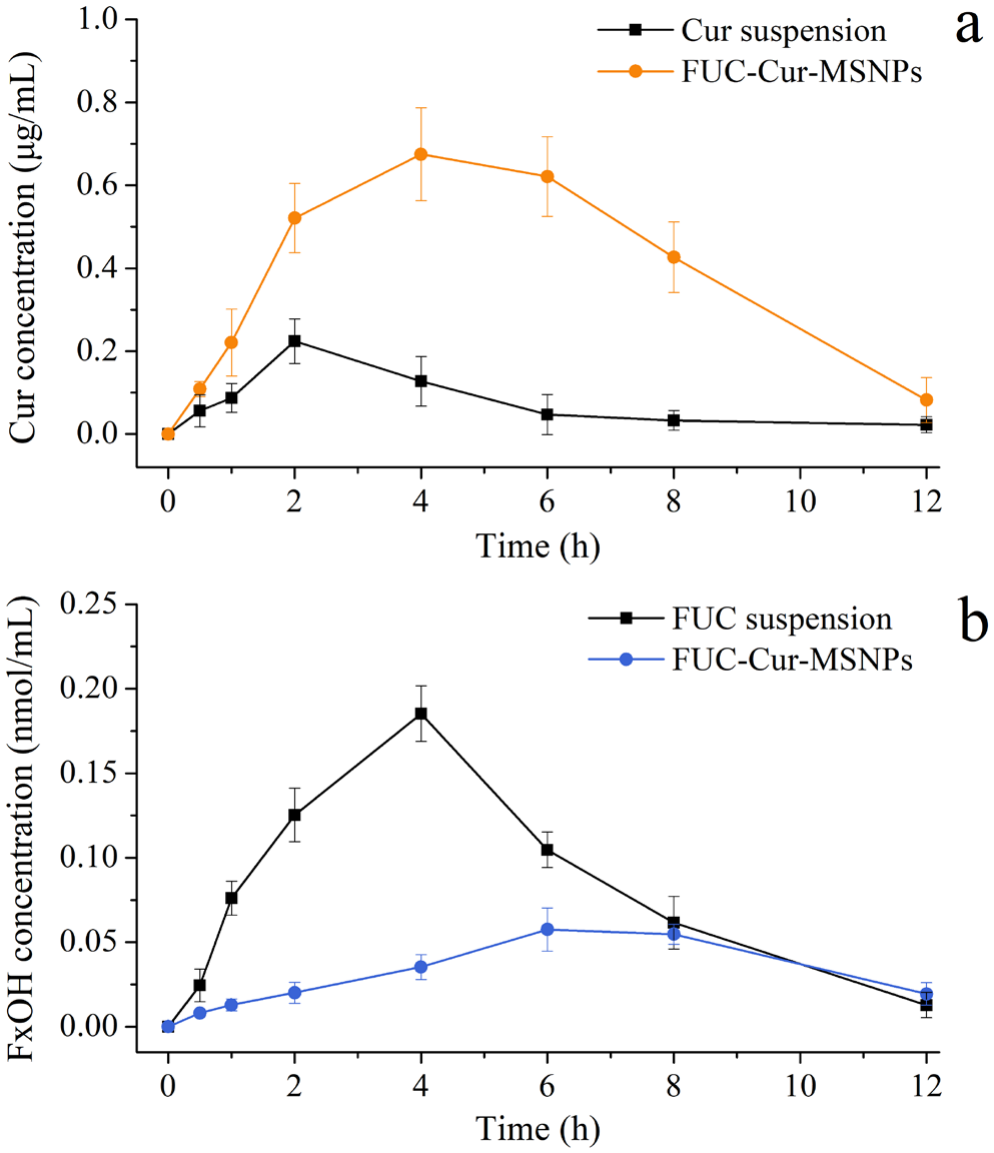
Plasma level‐time profiles of Cur (a) and FxOH (b) after oral administration of FUC‐Cur‐MSNPs.

In this section, the bioavailability of the encapsulated bioactives was evaluated by measuring the pharmacokinetic parameters in mice after oral gavage of FUC‐Cur‐MSNPs. Changes in plasma levels of the bioactives with time and their pharmacokinetic parameters are illustrated in Figure 7 and Table 2, respectively. The plasma concentration of Cur in the Cur suspension group rapidly reached a peak (*C*_max_, 0.224 μg/mL) at 2 h post‐administration and declined slowly thereafter, with a *T*_max_ value similar to that reported (Chavez‐Zamudio et al. 2017). The *C*_max_ of Cur in the FUC‐Cur‐MSNPs group was 0.675 μg/mL, which occurred 4 h after gavage, and the area under the curve (AUC_0–12_) was 5.037 h μg/mL. It is well known that the shorter the time to reach the absorption peak, the quicker the action will be, and a long *T*_max_ indicates slower absorption of the bioactives and a longer duration of action. By comparison, it was found that MSNPs increased the concentration of Cur entering the blood circulation and prolonged its duration of action. In addition, encapsulating Cur in MSNPs increased its bioavailability up to 4.7‐fold.

**TABLE 2:** Pharmacokinetic parameters of Cur and FxOH after oral administration of FUC‐Cur‐MSNPs.

Sample		*T*_max_	*C*_max_	AUC_0–12_	MRT_0–12_
Cur	Cur suspension	2	0.224	0.916	4.002
	FUC‐Cur‐MSNPs	4	0.675	5.037	5.323
FxOH	FUC suspension	4	0.185	1.048	4.739
	FUC‐Cur‐MSNPs	6	0.058	0.433	6.528

*Note:* The units of *T*_max_ and MRT_0–12_ are hours. The units of *C*_max_ for Cur and FxOH are μg/mL and nmol/mL, respectively. The units of AUC_0–12_ for Cur and FxOH are h μg/mL and h nmol/mL, respectively.

Pharmacokinetic analysis of FUC encapsulated in FUC‐Cur‐MSNPs was expressed as fucoxanthinol (FxOH) due to the susceptibility of the free state of FUC to be converted to the form of its metabolite, FxOH, by lipase, cholesterol esterase, and carboxyl esterase in the GIT (Liu et al. 2019). The *T*_max_ of the FUC suspension group was 4 h, consistent with previous reports (Koo et al. 2016). In contrast, the *T*_max_ in the FUC‐Cur‐MSNPs group was found to be extended to 6 h, indicating that embedding FUC in MSNPs dramatically prolonged its action time. The slow degradation of enzymes and microflora present in the colon leads to the prolonged release and action of bioactives encapsulated in a delivery system with colon‐targeted delivery properties (Rai et al. 2016), confirming the ability of MSNPs to target the delivery of bioactives encapsulated in the inner layers to the colon. The *C*_max_ of free FUC was up to 0.185 nmol/mL, whereas that of FUC encapsulated in MSNPs was only 0.058 nmol/mL. Most of the free state FUC could be absorbed in the upper GIT, while only a minimal amount of FUC was released into the small intestine due to the encapsulation of FUC‐Cur‐MSNPs, thus its hydrolytic transformation in the GIT and its entry into the blood circulation were low. Overall, MSNPs could effectively improve the bioavailability of bioactives encapsulated in the outer layer and reduce the biodistribution of the inner layer bioactives in the upper GIT.

## 4 Conclusions

In this paper, Gli‐CMK NPs with colon‐targeted delivery properties were used as the inner core, and CHC was used as the outermost carrier material to prepare a co‐delivery system based on MSNPs by the layer‐by‐layer self‐assembly technique, which realized the co‐encapsulation of Cur and FUC as well as the programmed sequential release of Cur and FUC in the small intestine and colon, respectively. In vitro digestion studies revealed that MSNPs maintained a relatively stable state in SGF. A small amount of Cur located in the outer layer of MSNPs was released through a concurrent mechanism of diffusion and erosion, while the release of FUC located in the inner layer was mainly dominated by diffusion. The structure of MSNPs was disrupted in SIF, where Cur was released in large quantities via the erosive mechanism. Due to the degradation of β‐mannanase in SCF, a large amount of FUC was released from exposed Gli‐CMK NPs by erosion. Furthermore, in vivo pharmacokinetic analyses confirmed that MSNPs enhanced the bioavailability of bioactives encapsulated in the outer layer, reduced the release of bioactives encapsulated in the inner layer in the upper GIT, and realized their delivery to the colon. The present study is valuable for expanding the application of MSNPs with programmed sequential release performances in the field of co‐delivery, as well as for improving the bioavailability of multiple bioactives. Furthermore, the MSNPs constructed in this study serve as a versatile strategy for programmed sequential release of other bioactive pairs with distinct site‐specific requirements in the GIT, beyond the co‐encapsulation of Cur and FUC.

## Author Contributions

**Luhui Wang:** conceptualization, methodology, software, data curation, investigation, formal analysis, project administration, funding acquisition, writing – original draft, writing – review and editing. **Mingqing Wang:** resources, writing – review and editing, project administration. **Ling Lv:** methodology, software, writing – review and editing. **Changhu Xue:** conceptualization, resources, supervision, funding acquisition, project administration.

## Funding

This work was supported by the Taishan Scholar Program of Shandong Province (Grant tsqnz20250761), Innovation Project of Agricultural Science and Technology of Shandong Academy of Agricultural Sciences (Grant CXGC2025F21‐1‐19), and Key R&D Program of Shandong Province (Grant 2023TZXD074).

## Ethics Statement

This study was approved by the Institutional Review Board of Ocean University of China.

## Conflicts of Interest

The authors declare no conflicts of interest.

## Data Availability

Data will be made available on request.
